# Comparative analysis of size and shape variation in *Tridacna maxima* (Röding, 1798) along the central and northern Red Sea coasts of Saudi Arabia

**DOI:** 10.7717/peerj.21589

**Published:** 2026-08-24

**Authors:** Abdelwaheb Ben-Othmen, Nur Zulfa Qalesya Thomas, Jongkar Grinang

**Affiliations:** 1 Laboratory of Biochemistry, LR12ES05 (Lab-NAFS) “Nutrition, Functional Foods and Vascular Health”, Faculty of Medicine of Monastir, Tunisia; 2 Institute of Biodiversity and Environmental Conservation, Universiti Malaysia Sarawak, Kuching, Sarawak, Malaysia

**Keywords:** Bivales, Geometric morphometric, Linear regression, Red Sea, Phenotypic plasticity

## Abstract

This study examines regional and genetic influences on shell morphology of the small giant clam *Tridacna maxima* from the central and northern Red Sea of Saudi Arabia using linear mixed model and geometric morphometrics. Shell height and length exhibit significant regional differences, with individuals from the North showing greater dimensions (height: F_1,17_ = 12.102, *p* = 0.003; length: F_1,17_ = 9.219, *p* = 0.007) than those from the Central region. No significant effects were observed for haplotype, side, or their interactions, suggesting that shell size variation reflects environmental rather than genetic drivers. Geometric morphometric analysis revealed that the first two principal components explained 44% of total shape variance, but discriminant analysis showed weak and nonsignificant group separation (Mahalanobis distance = 1.78–1.89; *p* = 0.999). The stability of shell form across haplotypes and regions indicates developmental canalization, with phenotypic plasticity influencing size under different environmental conditions. Larger shells in the North likely reflect more favourable hydrological and nutrient regimes, while smaller shells in the Central region may be linked to higher turbidity and anthropogenic stress. These findings highlight environmental modulation of clam growth in the Red Sea, supporting the view that regional habitat variability, rather than genetic differentiation, primarily shapes phenotypic diversity in *T. maxima* populations.

## Introduction

Morphometric variation can be used to discriminate phenotypic stocks, as the interactive effects of environment, selection and genetics produce morphometric differences within a species (Cadrin, 2000; Dwivedi, 2019; Muniz et al., 2020). Geometric morphometrics is a method employed to study shape especially shape of hard structures that can be based on a coordinate system (Bookstein, 1991; Zelditch et al., 2004). The efficiency of this morphometric method to evaluate shape variation and covariation of biological structures has been evidenced both at the inter- and intra-specific levels (Lima et al., 2014; Christe et al., 2019; Cerda et al., 2021). Indeed, consistency has been found between results of shape obtained by geometric morphometrics and molecular data at the intra-specific level in animal groups such as fish, insects and crustaceans (Tripp-Valdez et al., 2012; Valentin et al., 2014; Oleska & Tofilski, 2015; Grinang, Das & Ng, 2019; Tanawat et al., 2022; Jeon et al., 2023; Casaubon & Riehl, 2024). Moreover, due to their hard consistency, bivalve shells or valves are suitable structures to evaluate shape by geometric morphometrics (Morais et al., 2014), although these features have not been assessed in giant clams of the genus *Tridacna*.

The small giant clam *Tridacna maxima* (Röding, 1798) is member of subfamily Tridacninae (Bivalvia: Cardiidae), a cosmopolitan species in the Indo-West Pacific (Harzhauser et al., 2008) and representing the most abundant species of the genus *Tridacna* in the Red Sea (Pappas et al., 2017; Lim et al., 2020). It is among the smallest species of the genus *Tridacna*, typically reaching sexual maturity between 6 and 13 cm in shell length, although individuals may grow up to 41.7 cm (Palomares & Pauly, 2025). This species inhabits shallow coral reef environments, where it contributes to reef productivity and structural complexity through its symbiotic relationship with photosynthetic zooxanthellae. *T. maxima* is harvested for food and collected for the ornamental shell trade, and it supports small-scale commercial fisheries across much of its range. Although listed as Least Concern by the IUCN (Neo & Li, 2024), it remains included in CITES (Palomares & Pauly, 2025), highlighting the need for regulated trade to prevent overexploitation. The life history strategy of this species is not peculiar, though the long life-span is remarkable (100 years). *T. maxima* are hermaphrodites (Wada, 1954), and characterized by a short larval stage of 8–10 days (Jameson, 1976; Lucas, 1988). Its dispersal during the larval stage is strongly dependent on currents (Cowen & Sponaugle, 2009; Fauvelot et al., 2020).

These characteristics combined with its large distributional range have led researchers to conduct studies aiming at detecting population structure in different geographic areas of the small giant clam distribution (DeBoer et al., 2008, 2014; Kochzius & Nuryanto, 2008; Nuryanto & Kochzius, 2009; Hui et al., 2016; Neo et al., 2017; Pappas et al., 2017; Keyse et al., 2018; Fauvelot et al., 2020; Lim et al., 2020; Othmen et al., 2020; Lee et al., 2022). Othmen et al. (2020) evaluated the population structure of *T. maxima* based on the analysis of partial COI gene sequences of specimens collected along a large geographic area (660 km), covering the central and northeast coast of the Red Sea.

The study by Othmen et al. (2020) investigated how potential barriers to gene flow influence the genetic connectivity among populations of the relatively low-dispersal bivalve *Tridacna maxima* along the Saudi Arabian Red Sea coast. Forty-four specimens were collected from four localities (Wajh, Umluj, Yanbu, and Jeddah), and genetic variation was assessed using the highly variable mitochondrial cytochrome c oxidase I (CO1) marker. Although no significant population genetic structure was detected, the analysis identified two distinct mitochondrial haplogroups. Haplogroup 1 was moderately supported (posterior probability, PP = 0.89), whereas Haplogroup 2 showed weaker phylogenetic support (PP = 0.31). This mitochondrial pattern was further reflected in a statistical parsimony network displaying two star-like sub-networks centred around the common haplotypes H1 and H2 (Othmen et al., 2020).

Despite the pronounced environmental heterogeneity of the Red Sea, including latitudinal gradients in temperature and salinity, previous analyses based on mitochondrial CO1 markers did not detect clear population genetic structuring in *Tridacna maxima* along the Saudi Arabian Red Sea coast (Othmen et al., 2020). In contrast, population differentiation associated with environmental gradients has been reported in other Red Sea marine organisms, including the coral reef fish *Amphiprion bicinctus* and the reef sponge *Stylissa carteri*, using broader sampling designs and higher-resolution genetic markers (Nanninga et al., 2014; Giles et al., 2015). Nevertheless, Othmen et al. (2020) identified two distinct mitochondrial haplogroups in *T. maxima*, raising the unresolved question of whether these genetic groupings correspond to morphological differentiation. This represents an important knowledge gap, particularly because *T. maxima* is widely harvested, plays a significant role in coral reef ecosystems, and remains classified as Least Concern by the IUCN despite the limited availability of integrated population-level morphological and genetic assessments in the Red Sea. To address this gap, the present study applied geometric morphometric analyses to genetically characterized specimens to evaluate shell morphological variation among haplotypes and between populations from the northern and central Red Sea regions of Saudi Arabia.

## Materials and Methods

### Sample collection

**Figure 1:**
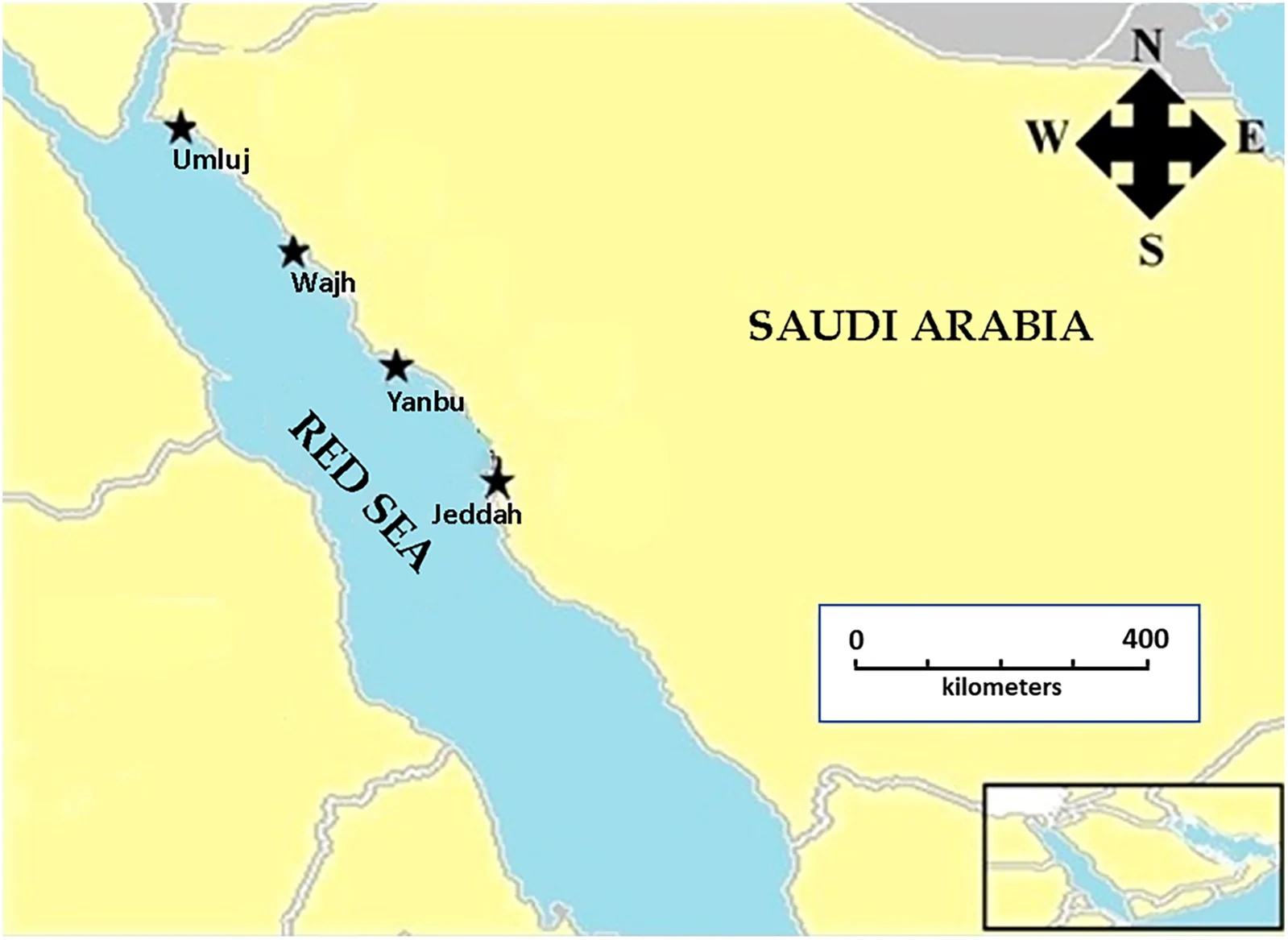
Sampling locations of the small giant clam *Tridacna maxima* across the Red Sea coast of Saudi Arabia.

**Figure 2:**
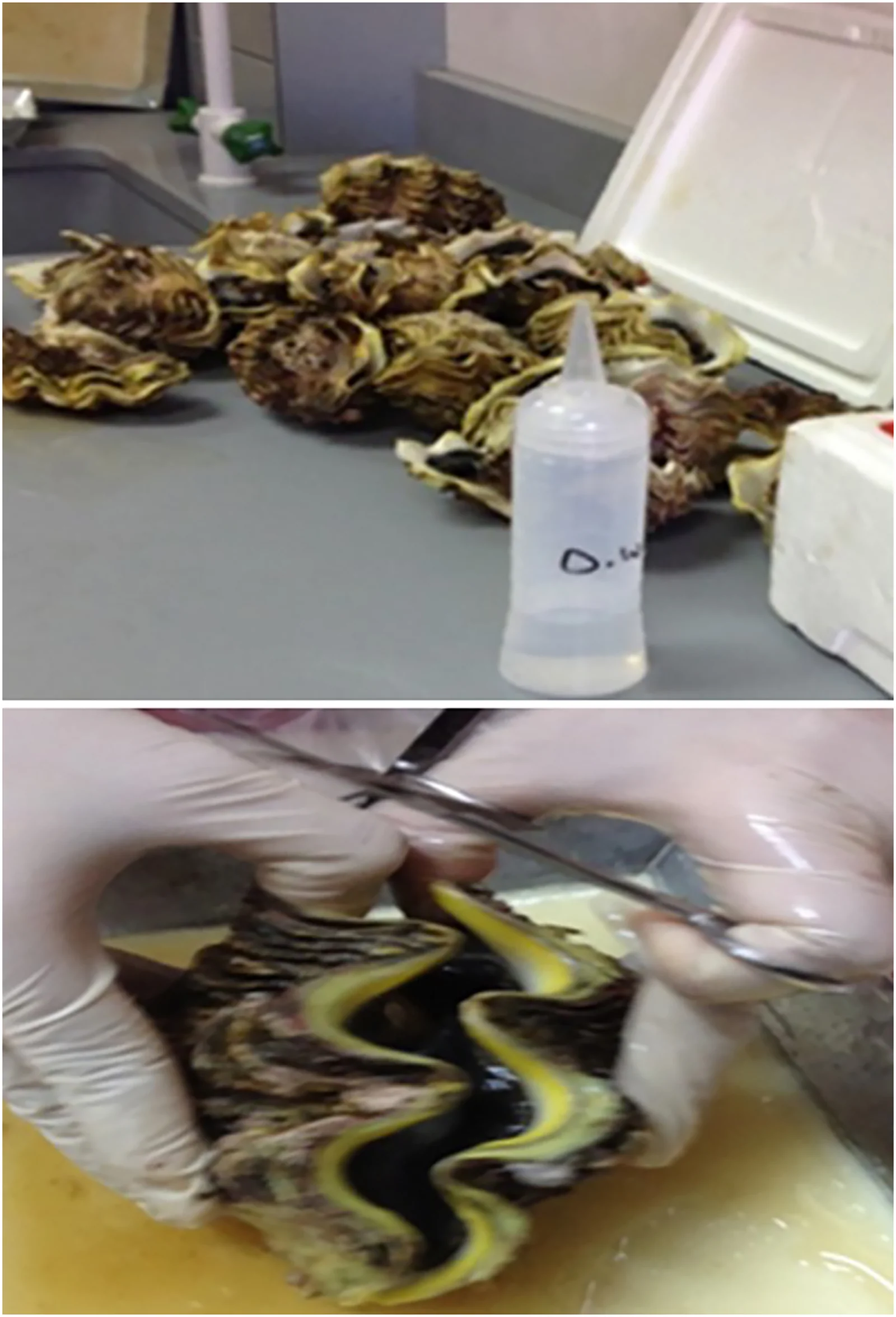
Example of small giant clam, *Tridacna maxima* samples used in the study.

The *Tridacna maxima* specimens used in this study were derived from the same collection examined by Othmen et al. (2020). However, only 21 of the 44 available specimens were included in the present analysis after evaluating specimen suitability and image quality for geometric morphometric analyses. The clams were collected from four localities along the Red Sea coast of Saudi Arabia, comprising two sites in the northern region (Wajh and Umluj) and two in the central region (Yanbu and Jeddah) (Figs. 1 and 2, Table 1). During field expeditions conducted in February and March 2014, specimens of small giant clams were collected from four reef sites along the Red Sea coast of Saudi Arabia (Fig. 1). Sampling was carried out using SCUBA at depths between 1 and 10 m. The surface GPS coordinates of each collection site were recorded. Specimens were identified as *Tridacna maxima* following Rosewater (1965). After removing the soft tissues for molecular analysis, the shells were cleaned in hot water and air-dried prior to morphometric measurements. Both the ventral and dorsal surfaces of each shell were photographed using a Canon EOS 600D digital camera under consistent distance and angle conditions. Shell height (SH in cm) was measured as the maximum dorsal–ventral dimension of the shell perpendicular to its length, while shell length (SL in cm) was defined as the maximum antero-posterior dimension. Each of the 21 individuals was measured on both sides, yielding a total of 42 observations (Table 1). No field permit is required for this study.

**Table 1:** Summary of 21 *Tridacna maxima* specimens collected from four localities along the Red Sea coast of Saudi Arabia.

No specimen	Haplotype	Region	Shell height (cm)		Shell length (cm)		GPS coordinates
			Left	Right	Left	Right	
22 Wajh	H1	North	5.92	6.61	10.08	9.15	N 26.28333, E 36.41667
24 Wajh	H1	North	7.90	7.51	14.63	15.85	N 26.28333, E 36.41667
26 Wajh	H1	North	7.53	8.10	11.38	11.16	N 26.28333, E 36.41667
27 Wajh	H1	North	8.46	7.29	13.14	13.28	N 26.28333, E 36.41667
28 Umluj	H1	North	7.78	8.07	11.20	10.89	N 25.03697, E 37.28778
30 Umluj	H1	North	7.53	6.43	14.29	15.73	N 25.03697, E 37.28778
31 Umluj	H1	North	11.45	12.38	14.58	16.29	N 25.03697, E 37.28778
33 Yanbu	H1	Central	4.72	4.60	8.61	7.14	N 24.08222, E 38.06556
36 Yanbu	H1	Central	6.24	5.60	8.76	7.93	N 24.08222, E 38.06556
39 Jeddah	H1	Central	6.68	6.02	9.81	11.06	N 22.28333, E 39.10000
23 Wajh	H2	North	7.05	6.95	10.03	10.37	N 26.28333, E 36.41667
25 Wajh	H2	North	6.92	6.48	12.53	13.23	N 26.28333, E 36.41667
29 Umluj	H2	North	8.29	8.76	8.54	7.07	N 25.03697, E 37.28778
32 Umluj	H2	North	9.15	9.91	10.40	10.30	N 25.03697, E 37.28778
34 Yanbu	H2	Central	5.80	5.63	7.31	7.07	N 24.08222, E 38.06556
35 Yanbu	H2	Central	6.19	5.58	7.78	8.98	N 24.08222, E 38.06556
37 Yanbu	H2	Central	6.07	5.72	8.39	8.95	N 24.08222, E 38.06556
40 Jeddah	H2	Central	6.51	6.58	9.22	9.20	N 22.28333, E 39.10000
41 Jeddah	H2	Central	6.78	6.63	9.30	9.86	N 22.28333, E 39.10000
42 Jeddah	H2	Central	6.41	6.90	10.67	11.35	N 22.28333, E 39.10000
43 Jeddah	H2	Central	6.21	6.51	9.88	9.93	N 22.28333, E 39.10000

### Linear mixed model

A linear mixed model (LMM) was used to examine whether shell height and length were significantly influenced by shell side (left/right), haplotype (H1/H2), and region (North/Central). In the model, side, haplotype, and region were treated as fixed factors, while individual clam was included as a random effect to account for repeated measurements within each specimen. This structure allowed the model to partition within-individual variation from among-individual differences and appropriately handle the non-independence of bilateral measurements. The LMM was fitted using restricted maximum likelihood estimation, and model significance was evaluated using F-tests with Kenward–Roger’s approximation for degrees of freedom. The analysis was performed in JASP Version 0.95 (JASP Team, 2025).

### Geometric morphometric analysis

**Figure 3:**
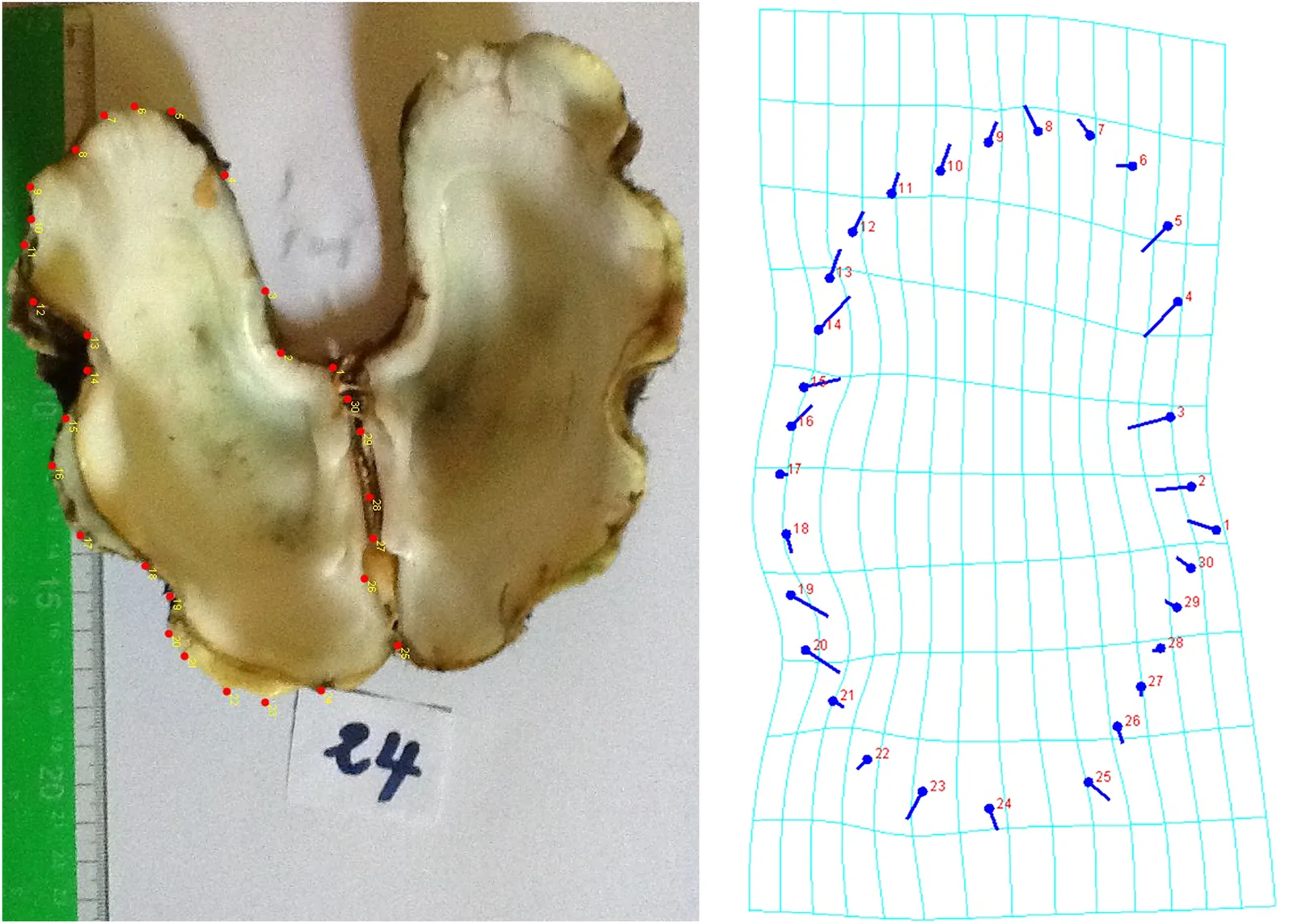
Thirty homologous landmarks digitized on the right valve of *Tridacna maxima*. The grid illustrates the spatial configuration of landmarks used in geometric morphometric analysis.

Geometric morphometric analysis (GMA) was performed to assess phenotypic variation in the clam and to determine whether shell shape differences were associated with haplotypic genotypes and sampling localities. To test this hypothesis, the right valve of each specimen was analyzed, comprising 10 individuals of haplotype H1 and 11 individuals of haplotype H2. The 21 clams were further classified by region, with 11 individuals from the northern sites and 10 from the central sites (Table 1). Digital images of the lateral internal view of each right valve were captured using a high-resolution camera under standardized lighting and orientation conditions (Fig. 3).

GMA began with the generation and processing of landmark data using tpsUtil (Rohlf, 2015) and tpsDig2 (Rohlf, 2018). A total of 30 homologous landmarks were digitized on each right valve to capture key morphological features associated with haplotype and regional variation (Fig. 2). The selected landmarks were chosen for their consistency and diagnostic value in representing shell shape differences among groups. The resulting landmark configurations were standardized using Generalized Procrustes Analysis (GPA) to remove variation due to size, position, and orientation (Bookstein, 1997; Zelditch et al., 2004). Subsequently, Principal Component Analysis (PCA) was performed to explore major axes of shape variation based on the covariance matrix of Procrustes coordinates. Discriminant Function Analysis (DFA) was then applied to test whether individuals could be reliably differentiated according to haplotype and region. Group dissimilarities were quantified using Mahalanobis distances and Hotelling’s T^2^ statistics, with 1,000 permutations conducted to assess statistical significance at *p* = 0.05. All analyses were conducted using MorphoJ version 1.08.02 (Klingenberg, 2011).

## Results

### Size variation

**Figure 4:**
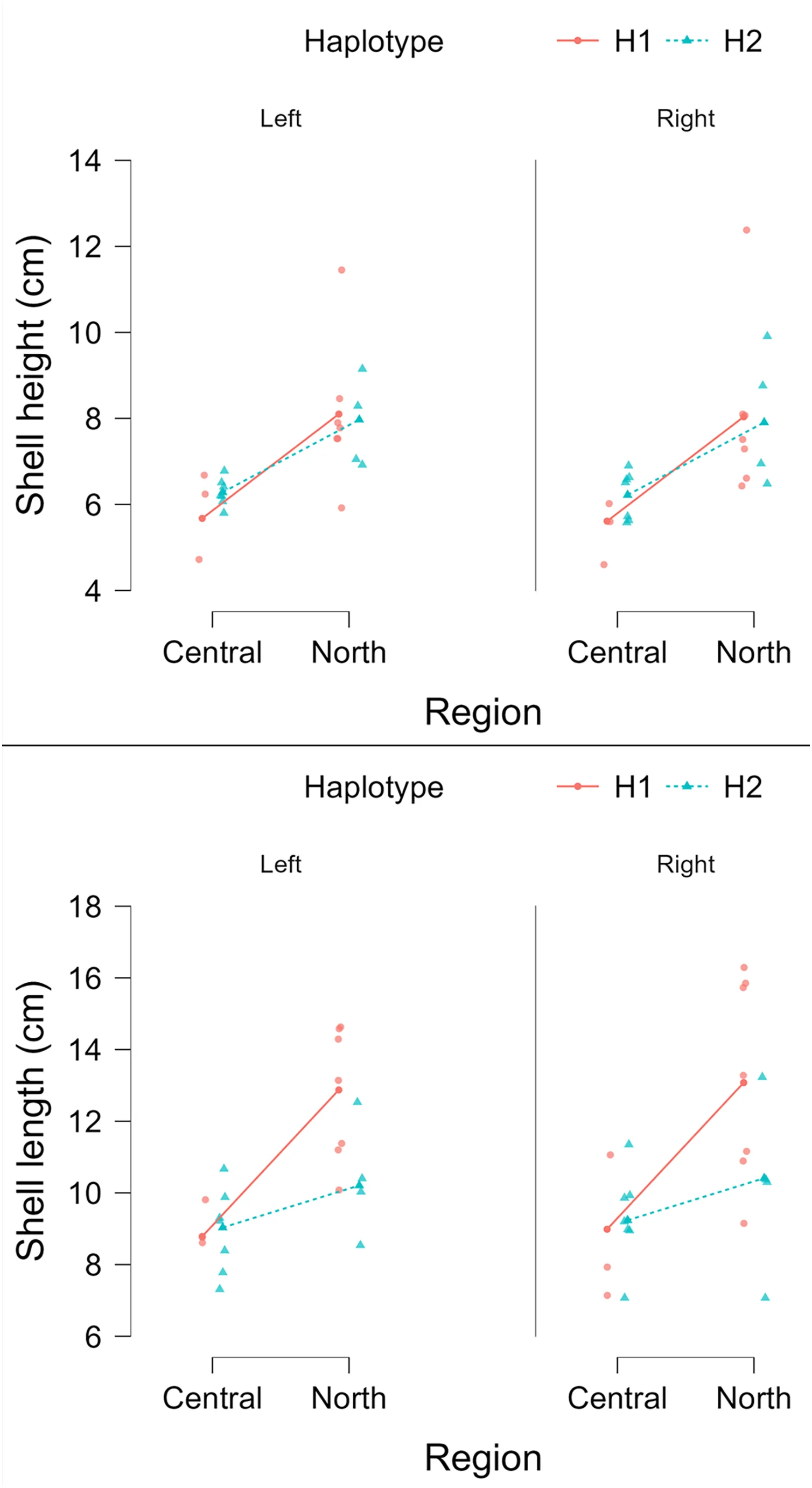
Plot of shell height and length of *Tridacna maxima* across haplotypes, regions, and sides.

The linear mixed model revealed a significant effect of region on shell height (F_1,17_ = 12.102, *p* = 0.003). Individual clams from the North region exhibited greater shell height (estimated marginal mean = 8.004 ± 0.397 cm, 95% Confidence Interval (CI) [7.225–8.783]) than those from the Central region (5.947 ± 0.438 cm, 95% CI [5.090–6.805]), corresponding to an average difference of approximately 2.06 cm. In contrast, side (F_1,20_ = 0.236, *p* = 0.632), haplotype (F_1,17_ = 0.164, *p* = 0.691), and the haplotype × region interaction (F_1,17_ = 0.390, *p* = 0.541) did not significantly influence shell height (Tables 2–4, Fig. 4).

**Table 2:** Summary of linear mixed model analysis evaluating the effects of mitochondrial CO1 haplotype, region, shell side, and their interaction on shell height and shell length of *Tridacna maxima*.

Effect	df	Shell height (cm)		Shell length (cm)	
		F	*p*	F	*p*
Side	1, 20	0.236	0.632	1.052	0.317
Haplotype	1, 17	0.164	0.691	1.943	0.181
Region	1, 17	12.102	0.003	9.219	0.007
Haplotype × Region	1, 17	0.390	0.541	2.830	0.111

**Table 3:** Estimated marginal means for shell height and shell length of *Tridacna maxima* from the North and Central regions of the Red Sea obtained from linear mixed model analysis.

	Estimate	SE	95% confidence interval	
			Lower	Upper
**Shell height (cm)**				
North	8.004	0.397	7.225	8.783
Central	5.947	0.438	5.090	6.805
**Shell length (cm)**				
North	11.642	0.583	10.500	12.780
Central	9.010	0.642	7.752	10.270

**Table 4:** Fixed-effect estimates from the linear mixed models for predictor variables influencing shell morphology in *Tridacna maxima*. The reference levels for side, haplotype, and region are the right side, H1, and North region, respectively.

	Estimate	SE	df	*t*	*p*
**Shell height (cm)**					
Intercept	6.976	0.296	17	23.603	<0.001
Side	0.032	0.065	20	0.486	0.632
Haplotype	−0.120	0.296	17	−0.405	0.691
Region	−1.028	0.296	17	−3.479	0.003
Haplotype × Region	−0.184	0.296	17	−0.624	0.541
**Shell length (cm)**					
Intercept	10.326	0.433	17	23.826	<0.001
Side	−0.101	0.099	20	−1.026	0.317
Haplotype	0.604	0.433	17	1.392	0.181
Region	−1.316	0.433	17	−3.036	0.007
Haplotype × Region	−0.729	0.433	17	−1.682	0.111

A similar pattern was observed for shell length, with region exerting a significant effect (F_1,17_ = 9.219, *p* = 0.007). Individual clams from the North region had greater shell length (11.642 ± 0.583 cm, 95% CI [10.500–12.780]) than those from the Central region (9.010 ± 0.642 cm, 95% CI [7.752–10.270]), representing an average difference of approximately 2.63 cm. No significant effects were detected for side (F_1,20_ = 1.052, *p* = 0.317), haplotype (F_1,17_ = 1.943, *p* = 0.181), or the haplotype × region interaction (F_1,17_ = 2.830, *p* = 0.111). Collectively, these results suggest that regional environmental conditions play a stronger in shaping shell morphology than mitochondrial haplotype variation, with consistent bilateral symmetry and no evidence of genotype by region interaction (Tables 2–4, Fig. 4). Nevertheless, the lack of significant haplotype effects should be interpreted cautiously, as the current analysis was based on CO1-defined mitochondrial haplotypes and relatively limited sample sizes, which may reduce the ability to detect subtle genetic contributions to shell morphological variation.

### Shape variation

**Figure 5:**
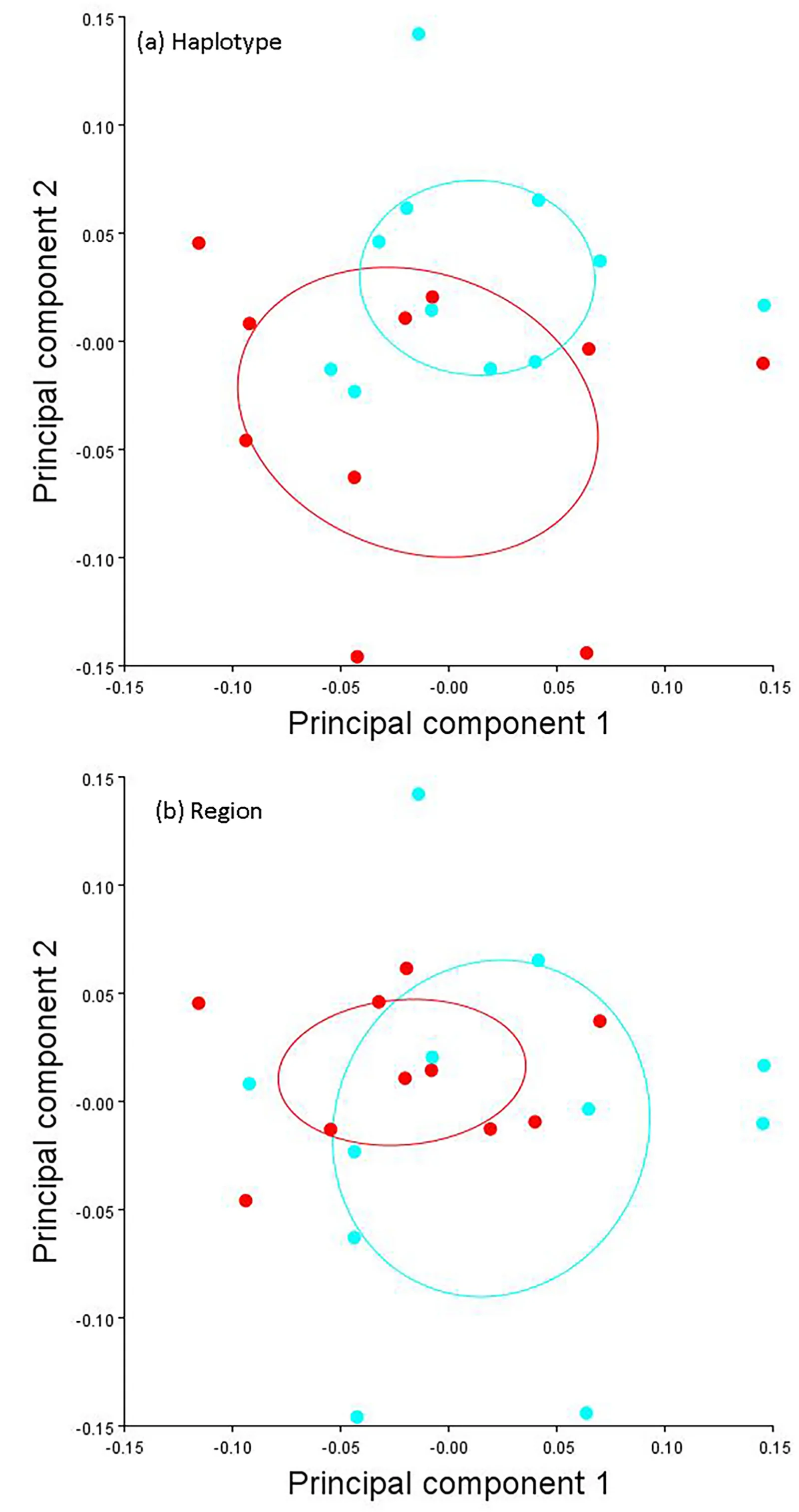
Principal component analysis (PCA) biplot of right valve shape in *Tridacna maxima* specimens from the Red Sea, showing overlapping morphotypes. (A) Region, (B) Haplotype.

**Figure 6:**
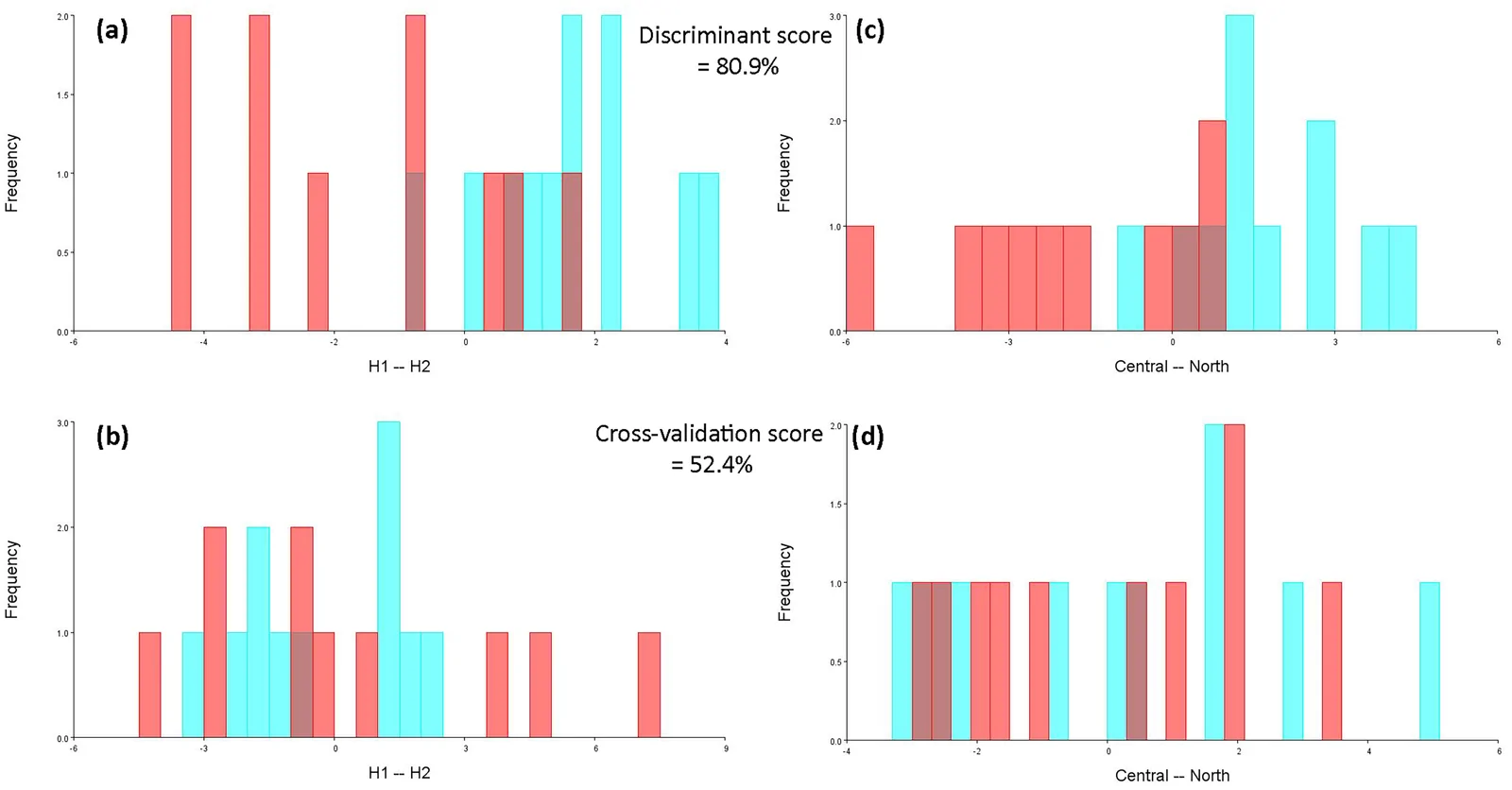
Discriminant and cross-validation scores for (A, B) haplotypes and (C, D) regions.

Geometric morphometric analysis revealed that the first two principal components accounted for 44% of total shell shape variance (PC1 = 24.0%, PC2 = 20.0%), reflecting moderate but continuous shape variation among individuals (Table 5, Fig. 5). Discriminant function analysis showed weak separation between both haplotypes and regions (Mahalanobis distance = 1.78–1.89; *p* = 0.999), with original classification accuracy of 80.9% but a substantially lower cross-validation rate of 52.4% (Table 5, Fig. 6). These results indicate that although some variation in shape exists, it is not statistically significant nor consistent enough to distinguish groups. When considered alongside the linear mixed model results, which showed significant regional differences in shell size but not haplotype effects, it appears that environmental conditions primarily influence overall shell growth rather than geometric shape. The morphological stability across haplotypes and regions suggests developmental canalization of shell form, with size plasticity reflecting ecophenotypic responses to local environmental gradients rather than genetic differentiation.

**Table 5:** Summary of principal component analysis (PCA) and discriminant function analysis (DFA).

Analysis	Haplotype	Region
**Principal component analysis**		
PC1	24.0%	
PC2	20.0%	
**Discriminant function analysis**		
Mahalanobis distance	1.784	1.886
T^2^, *p*-value	16.681, 0.999	18.625, 0.999
Overall discriminant score	80.9%	80.9%
Cross-validation score	52.4%	52.4%

## Discussion

The significant regional differences in both shell height and length suggest that environmental rather than genetic factors are the primary drivers of morphological variation in these small giant clams. Individuals from the North region exhibited consistently larger shells, implying more favourable growth conditions, possibly linked to higher nutrient availability, better water circulation, or reduced sedimentation stress. In contrast, the smaller shell dimensions observed in the Central region may reflect environmental constraints such as elevated turbidity, lower productivity, or increased anthropogenic disturbance. The absence of haplotype effects indicates that the observed morphological disparity is not genetically based but rather a phenotypic response to local environmental conditions. This pattern aligns with previous findings that spatial heterogeneity in water quality and substrate composition can markedly influence growth performance and shell morphology in sessile or sedentary bivalves (Fassatoui, Shaiek & Romdhane, 2024; Liu et al., 2024). Overall, these results highlight the ecological significance of regional habitat conditions in shaping growth dynamics and support the inclusion of environmental variables in future morphometric or population genetic assessments. Nevertheless, future studies incorporating larger sample sizes across broader spatial and environmental gradients would further strengthen the robustness and generalizability of the present findings.

The phenotype of an organism can be modified through non-genetic processes collectively referred to as phenotypic plasticity, including acclimation and acclimatization. These responses may occur within a generation through reversible or developmental acclimation, or across generations through transgenerational effects mediated by parental provisioning, hormones, proteins, and epigenetic mechanisms (Munday, 2014). In contrast, genetic adaptation occurs across generations when heritable genetic changes confer favourable traits under specific environmental conditions. Both phenotypic plasticity and genetic adaptation are likely to occur in giant clams (Neo & Todd, 2011), however, their life-history characteristics suggest that these processes may operate over longer timescales compared with many other coral reef organisms. Thus, phenotypic plasticity may facilitate shorter- to medium-term responses to environmental change, whereas genetic adaptation is more likely to contribute to long-term evolutionary responses.

Shell morphology is known to exhibit substantial phenotypic plasticity in many mollusc species, often in response to predator-derived waterborne chemical cues (kairomones) (Brown, Eisner & Whittaker, 1970; Leonard, Bertness & Yund, 1999). Ling et al. (2008) demonstrated that the scutes of the fluted giant clam, *Tridacna squamosa*, function as an effective defence against crushing predation. This protective effect is achieved by increasing the apparent shell dimensions, thereby reducing the range of predators capable of grasping or crushing the clam. They further suggested that scute development may represent a less energetically costly defensive strategy than shell thickening or overall shell expansion, as enlarged shell dimensions can be attained more rapidly through scute formation than through shell growth alone.

In this study we evaluated the shape of right valve of small giant clam *T. maxima* obtained by geometric morphometrics and found that the variation of right valve shape from the central and northern east coast of the Red Sea is not consistent with the two haplogroups detected molecularly by Othmen et al. (2020) for the same clam specimens. While we evaluated the valve shape of small giant clams collected in an important portion of the east coast of the Red Sea (>600 km), based on molecular analyses, other authors confirmed that *T. maxima* is a panmictic population in the entire area (Lim et al., 2020). The shape of hard structures of marine organisms can reflect differences in environmental conditions and geographic locations, as these factors are well known to alter phenotypic traits (Rohlf & Marcus, 1993; Okoshi & Sato-Okoshi, 1996; Rice, 2012).

At high temperatures, giant clams undergo the same oxidative stress and bleaching as corals (Schwartzmann et al., 2011). They are further vulnerable to turbidity brought on by eutrophication (Hean & Cacho, 2003). Giant clams may exhibit reduced growth in the present of the same rapid industrial expansion in the 20th century led to coral loss in the Gulf of Aqaba (Northern Red Sea) because of rising nitrate, phosphate, and trace metal concentrations (Loya, 2004). Factors that are hurting the corals in the Northern Red Sea also affect them (Killam, Al-Najjar & Clapham, 2021).

In this sense, the Red Sea is characterized by strong latitudinal temperature and salinity gradients (Sofianos, Johns & Murray, 2002; Raitsos et al., 2013; Kürten et al., 2014), and it would be expected that the valve shape of the specimens evaluated here would concomitantly be discriminated in the presence of a given population structuring according to these gradients. Watters, Hoggarth & Stansbery (2009) reported that many unionoid mussels exhibit phenotypic plasticity that may be influenced by environmental factors like temperature, depth, and water flux as well as ecological parameters including population density, predation, and the quality and amount of food. According to Morais et al. (2014), water velocity is the main hydrological variable that influences shell morphology in unionoid mussels. According to various authors, the primary elements that can lead to modifications to shell shape are hydrological conditions and substrate composition (Hornbach, Kurth & Hove, 2010; Demayo, Cabacaba & Torres, 2012; Guarneri et al., 2014).

In our results, the right valve shape was not differentiated into two morphotypes, and no valve shape variation was detected in terms of the putatively differential oceanographic conditions among locations. In this sense, *Diplodon chilensis* (Bivalvia, Hyriidae) has different shell shapes depending on the site, according to research by Yusseppone et al. (2017). The shells from the Chimehuin River are longer, with the anterior part extending upward and the posterior part downward, and they have a steeper anterior curvature at the umbo than the shells from Paimn Lake. This could indicate that, despite exhibiting a short larval stage (Jameson, 1976; Lucas, 1988), the ocean current patterns that influence the central and north east Red Sea enable a very large dispersal (>600 km) of closely related individuals of *Tridacna maxima*. Because small giant clams are long-lived organisms, their shell morphology may reflect cumulative environmental influences over extended periods rather than only present-day conditions. Consequently, ongoing environmental changes in the Red Sea, including rising sea temperatures and alterations in water quality, could contribute to the morphological variation observed in this study. Given that *Tridacna maxima* is listed under CITES, understanding regional variation in shell growth may provide valuable baseline information for conservation and management strategies. Populations exhibiting larger shell sizes and more favourable growth conditions may contribute disproportionately to reef productivity and reproductive output, highlighting their ecological importance. Furthermore, recognising spatial variation in growth performance could support future efforts to develop region-specific monitoring, conservation, and sustainable management frameworks for small giant clam populations.

## Conclusion

This study demonstrates that regional environmental variability is more strongly associated with shell morphological variation in *Tridacna maxima* along the Red Sea than the mitochondrial haplotype groups examined in this study. Significant differences in shell height and length between the northern and central regions indicate that environmental factors such as nutrient availability, hydrodynamics, and sedimentation may strongly influence growth performance. In contrast, the limited geometric shape differentiation observed across regions and haplotypes suggests that shell shape remains comparatively stable, while shell size is more responsive to local environmental conditions. Although no significant haplotype effects were detected, this should not be interpreted as evidence for the absence of genetic influences on shell morphology, as the present analysis was based on mitochondrial CO1 haplotypes and relatively limited sample sizes. Nevertheless, the findings are consistent with the panmictic population structure previously reported for *T. maxima* and highlight the species’ capacity to persist across spatially heterogeneous habitats. Given the strong latitudinal gradients in temperature, salinity, and productivity along the Red Sea, future research should expand sampling to include southern populations, larger sample sizes, and higher-resolution genomic markers, together with finer-scale environmental measurements, to better resolve the relative contributions of environmental and genetic factors to shell morphological variation. Overall, shell morphology remains a useful but environmentally sensitive indicator of habitat quality and can complement molecular approaches in assessing the ecological and adaptive dynamics of this ecologically significant coral reef bivalve.

## Supplemental Information

10.7717/peerj.21589/supp-1 Supplemental Information 1 List of specimens used in the study.

10.7717/peerj.21589/supp-2 Supplemental Information 2 List of specimens used in the study, and the sampling sites.

10.7717/peerj.21589/supp-3 Supplemental Information 3 Sample 1: Haplotype 1 from Wajh, northern Red Sea.

10.7717/peerj.21589/supp-4 Supplemental Information 4 Sample 2: Haplotype 2 from Wajh, northern Red Sea.

10.7717/peerj.21589/supp-5 Supplemental Information 5 Sample 3: Haplotype 1 from Wajh, northern Red Sea.

10.7717/peerj.21589/supp-6 Supplemental Information 6 Sample 4: Haplotype 2 from Wajh, northern Red Sea.

10.7717/peerj.21589/supp-7 Supplemental Information 7 Sample 5: Haplotype 1 from Wajh, northern Red Sea.

10.7717/peerj.21589/supp-8 Supplemental Information 8 Sample 6: Haplotype 1 from Wajh, northern Red Sea.

10.7717/peerj.21589/supp-9 Supplemental Information 9 Sample 7: Haplotype 1 from Umluj, northern Red Sea.

10.7717/peerj.21589/supp-10 Supplemental Information 10 Sample 8: Haplotype 2 from Umluj, northern Red Sea.

10.7717/peerj.21589/supp-11 Supplemental Information 11 Sample 9: Haplotype 1 from Umluj, northern Red Sea.

10.7717/peerj.21589/supp-12 Supplemental Information 12 Sample X (left): Haplotype 1 from Umluj, northern Red Sea.

10.7717/peerj.21589/supp-13 Supplemental Information 13 Sample Y (right): Haplotype 1 from Umluj, northern Red Sea.

10.7717/peerj.21589/supp-14 Supplemental Information 14 Sample 11: Haplotype 2 from Umluj, northern Red Sea.

10.7717/peerj.21589/supp-15 Supplemental Information 15 Sample 12: Haplotype 1 from Yanbu, central Red Sea.

10.7717/peerj.21589/supp-16 Supplemental Information 16 Sample 13: Haplotype 2 from Yanbu, central Red Sea.

10.7717/peerj.21589/supp-17 Supplemental Information 17 Sample 14: Haplotype 2 from Yanbu, central Red Sea.

10.7717/peerj.21589/supp-18 Supplemental Information 18 Sample 15: Haplotype 1 from Yanbu, central Red Sea.

10.7717/peerj.21589/supp-19 Supplemental Information 19 Sample 16: Haplotype 2 from Yanbu, central Red Sea.

10.7717/peerj.21589/supp-20 Supplemental Information 20 Sample Z: Haplotype 1 from Rabagh, central Red Sea.

10.7717/peerj.21589/supp-21 Supplemental Information 21 Sample 17: Haplotype 1 from Jeddah, central Red Sea.

10.7717/peerj.21589/supp-22 Supplemental Information 22 Sample 18: Haplotype 2 from Jeddah, central Red Sea.

10.7717/peerj.21589/supp-23 Supplemental Information 23 Sample 19: Haplotype 2 from Jeddah, central Red Sea.

10.7717/peerj.21589/supp-24 Supplemental Information 24 Sample 20: Haplotype 2 from Jeddah, central Red Sea.

10.7717/peerj.21589/supp-25 Supplemental Information 25 Sample 21: Haplotype 2 from Jeddah, central Red Sea.

## Additional Information and Declarations

### Data Availability

The following information was supplied regarding data availability:

The raw data is available in the Supplemental Files.
