# Supplementary material for: Comparative analysis of size and shape variation in Tridacna maxima (Röding, 1798) along the central and northern Red Sea coasts of Saudi Arabia
Source: PeerJ. 2026 Aug 24;14:e21589. doi: 10.7717/peerj.21589 (PMC13525753; doi:10.7717/peerj.21589)
Supplement: Supplementary file 1 [file peerj-14-21589-s002.rtf]

H1: 22, 24, 26, 27, 28, 30, 31, 33, 36, 38, 39
Image			Site of sampling 	Geographic coordinates 	COI haplotype
28, 30, 31                           Umluj			25°02′13″N, 37°17′16″E		H1
22, 24, 26, 27                     Wajh			26°17′00″N, 36°25′00″E		H1
33, 36                                Yanbu			24°04′56″N, 38°03′56″E		H1
38                                        Rabagh		22°47′34″N, 39°02′01″E		H1
39                                        Jeddah		22°17′00″N, 39°06′00″E		H1
H2: 23,25,29,32,34,35,37,40,41,42,43
29, 32 			Umluj			25°02′13″N, 37°17′16″E		H2
23, 25 			Wajh			26°17′00″N, 36°25′00″E		H2
34, 35, 37 		Yanbu			24°04′56″N, 38°03′56″E		H2
40, 41, 42, 43 		Jeddah			22°17′00″N, 39°06′00″E		H2
